# Long-term follow-up of epidermolysis bullosa in real practice shows stability of the Epidermolysis Bullosa Disease Activity and Scarring Index and improvement with some off-label therapies

**DOI:** 10.1016/j.jdin.2022.07.008

**Published:** 2022-09-05

**Authors:** Genevieve Ho, Matthew Gibson, Nooshafarin Kazemikhoo, Dedee F. Murrell

**Affiliations:** a Department of Dermatology, St George Hospital, Sydney, Australia; b University of New South Wales, Faculty of Medicine and Health, Sydney, Australia; c The George Institute for Global Health, Sydney, Australia

**Keywords:** bullous disease, disease severity, epidermolysis bullosa, hereditary blistering disorders, interventions, outcomes, photobiomodulation

*To the Editor*: Epidermolysis bullosa (EB) is a rare, genetic, blistering disease that has no specific treatments. Trials are underway to compare new treatments with supportive care. Despite this, there is a lack of long-term objective data on how lesions change over time. We have used the validated Epidermolysis Bullosa Disease Activity and Scarring Index (EBDASI) at each visit of our patients with EB since 2013.^1^ The activity subscore tracks modifiable disease activity (blisters/erosions/crusting), whereas the damage subscore measures chronic components. Here, we summarize the disease trajectory in our patients.

Patients had EB simplex, dominant dystrophic epidermolysis bullosa, junctional epidermolysis bullosa (JEB), or recessive dystrophic epidermolysis bullosa (RDEB) (Table I). Eighteen patients who had at least 1 year of follow-up and 3 EBDASI assessments were included.

**Table I:** Baseline characteristics based on disease type

Subgroup		*N* (%)	Female, *n* (%)	Age range (y)	Total follow-up duration of patient (mo)	Mean follow-up duration (mo)	Range of follow-up duration (mo)
EBS	4		2 (50.0)	12-58	139	34.75	29-48
DDEB	3		1 (33.3)	25-72	124	41.3	15-60
JEB	5		1 (20.0)	18-47	275	55.0	9-97
RDEB	6		4 (66.7)	8-66	217	36.2	15-96
Total	18		8 (44.4)	2-72	755	39.4	9-97

*DDEB,* Dominant dystrophic epidermolysis bullosa; *EBS,* epidermolysis bullosa simplex; *JEB,* junctional epidermolysis bullosa; *RDEB,* recessive dystrophic epidermolysis bullosa.

EBDASI scores were generally lower in the 4 patients with EB simplex (Supplementary Fig 1, available via Mendeley at https://doi.org/10.17632/89jt9s77v8.2). Patient Australasian Epidermolysis Bullosa Registry (AEBR) #34 with comorbid atopic dermatitis had both the EBDASI and Eczema Area and Severity Index scores increasing with eczema flares (Fig 1, *A*, *green line*), suggesting that pruritus and scratching worsened his trauma-induced blisters (Fig 1, *A*). Patient AEBR#213 with dominant dystrophic epidermolysis bullosa (Fig 1, *B*) had a course of photobiomodulation, which is a low-level laser treatment that is believed to promote tissue healing and regeneration, and adenosine triphosphate, nucleic acid, and collagen synthesis.^2^ It has been reported to be effective in EB in a case report^2^ and an open-label study without the use of an objective score.^3^ Our patient received 21 sessions, 3 times a week in the first 4 weeks and then twice weekly (left side using red light, 650 nm, 6 J/cm^2^; right side using infrared light, 980 nm, 6 J/cm^2^; gums using red light, 650 nm, 1 J/cm^2^). The EBDASI activity score reduced from 53 to 37 and the damage score from 42 to 28 (exhibiting a minimal clinically important difference of 3). Five patients with JEB showed stable EBDASI scores over 54 visits over 275 months (Supplementary Fig 3, available via Mendeley at https://doi.org/10.17632/89jt9s77v8.2). An improved damage score was recorded for patient AEBR #11-1 with JEB (Fig 1, *C*) with the use of thalidomide. However, he was lost to follow-up and later died from a JEB-related cardiomyopathy. The use of colchicine and dapsone also show improvement in the EBDASI scores in JEB.^4^^,^^5^

**Fig 1:**
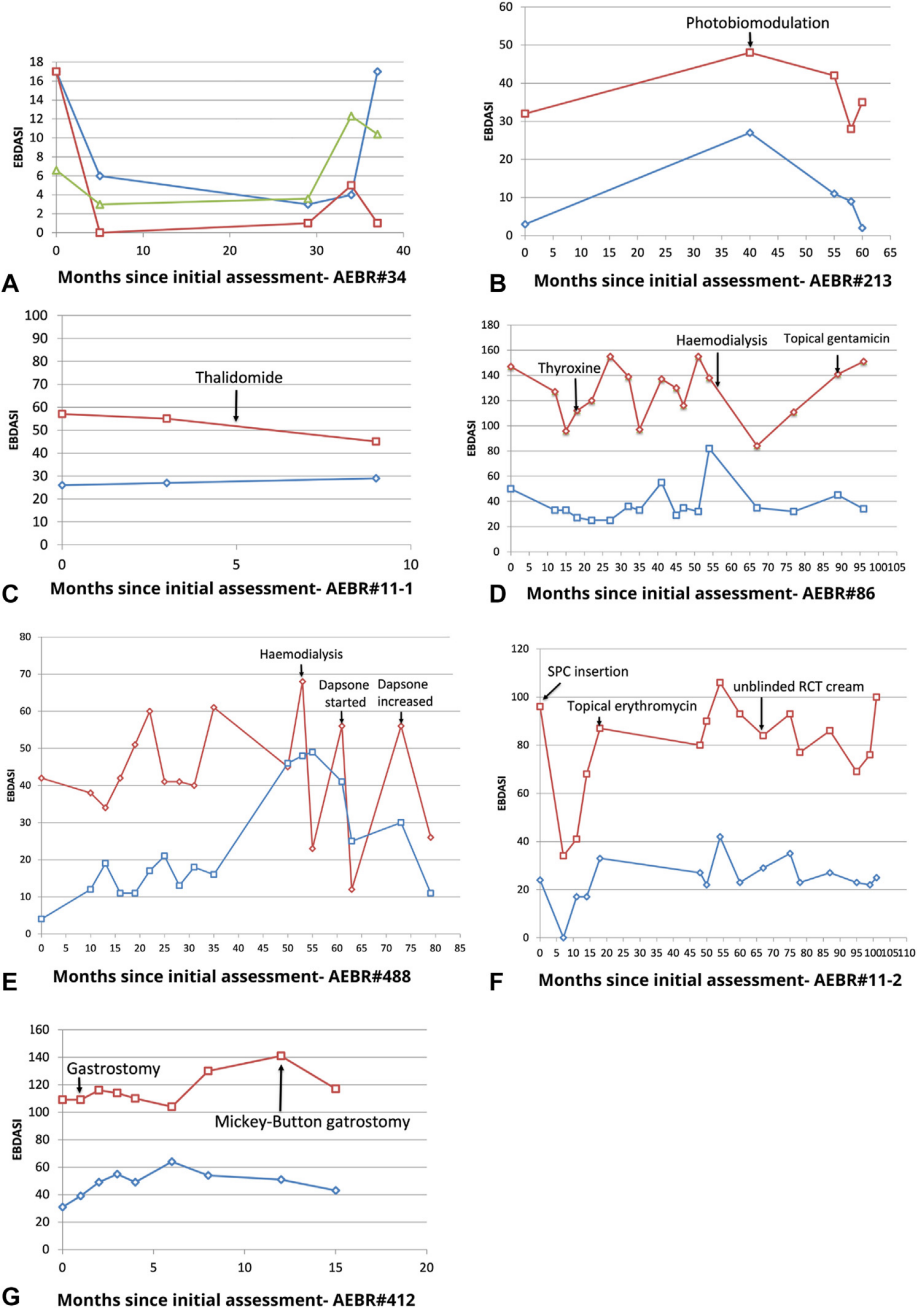
EBDASI scores of patients who underwent interventions (indicated by *arrows*) resulting in changes in EBDASI scores. (*blue line* = EBDASI activity subscores; *red line* = EBDASI damage subscores; *green line* = Eczema Area and Severity Index score.) *EBDASI,* Epidermolysis Bullosa Disease Activity and Scarring Index; *SPC*, suprapubic catheter; *RCT*, randomized control trial.

Six patients with RDEB (Supplementary Fig 4, available via Mendeley at https://doi.org/10.17632/89jt9s77v8.2) had stable EBDASI activity and damage scores over 217 months. In patient AEBR#86 (Fig 1, *D*), hemodialysis showed the biggest improvement, similar to that seen in patient AEBR#488 with JEB (Fig 1, *E*). Other interventions such as suprapubic catheter insertion in JEB (patient AEBR#11-2; Fig 1, *F*) and gastroscopy/percutaneous endoscopic gastrostomy feeding in RDEB (patient AEBR#412; Fig 1, *G*) resulted in some improvement in the EBDASI scores.

To our knowledge, this is the first observational report from real-world data documenting the long-term follow-up of EB using EBDASI. The overall trajectory of a patient’s disease activity remains stable over many years, with gradual increases in the absence of intervention, particularly for RDEB and JEB for which long-term scarring complications occur. How the disease affects each patient is highly varied; therefore, it is important to observe the trajectory of such scores rather than isolated values. The current report depicts the clinically significant impact of medical interventions and emerging therapies such as photobiomodulation, and the need for ongoing use of the EBDASI for future interventions and clinical trials.

## Conflicts of interest

The Australasian Blistering Diseases Foundation owns the license for the Epidermolysis Bullosa Disease Activity and Scarring Index and the Quality of Life in Epidermolysis Bullosa (QOLEB) tools. Drs Ho, Gibson, Kazemikhoo, and Murrell have no conflicts of interest to declare.
